# Research progress on plant-derived natural compounds regulating the MAPK signaling pathway for the prevention and therapy of Alzheimer’s disease

**DOI:** 10.3389/fphar.2025.1666082

**Published:** 2025-09-19

**Authors:** Xinyue Zhang, Shujia Huang, Hongfeng Xu, Yanan Hu, Ling Gao

**Affiliations:** Changchun University of Chinese Medicine, Changchun, China

**Keywords:** Alzheimer’s disease, neurodegenerative disease, natural compound, mitogen-activatedprotein kinase, research progress

## Abstract

Alzheimer’s disease (AD) is a progressive neurodegenerative disorder. It is characterised by the following: amyloid-β (Aβ) deposition, tau hyperphosphorylation, neuroinflammation and oxidative stress. Unfortunately, there is no curative treatment available. Recently, natural products have attracted growing interest as potential therapeutic agents for AD, thanks to their multi-target actions and favourable safety profiles. This review highlights recent advances in the use of various natural compounds, including flavonoids, phenolic compounds, saponins, terpenoids, alkaloids and coumarins, with a particular focus on how they modulate the mitogen-activated protein kinase (MAPK) signaling pathway. Representative agents such as myricetin, nobiletin, resveratrol, gallic acid, paeoniflorin, ganoderic acid A, huperzine A, triptolide, berberine, crocin, and ginsenosides have been shown to regulate MAPK subpathways (ERK, JNK, p38), thereby attenuating oxidative stress, neuroinflammation, synaptic dysfunction, and neuronal apoptosis. Preclinical studies suggest that these compounds improve cognitive function and ameliorate AD-related pathology, thereby supporting the idea that MAPK signaling is a critical therapeutic target. Nevertheless, current evidence is limited by short-term animal experiments, insufficient toxicological evaluations, and challenges related to bioavailability and blood–brain barrier penetration. Future studies should emphasize long-term efficacy, safety assessments, optimized drug delivery systems, and high-quality clinical trials. Overall, natural products represent a valuable source for AD drug discovery, and targeting MAPK signaling offers promising opportunities for novel therapeutic development.

## 1 Introduction

Since Alois Alzheimer first described it in 1907 (Alzheimer, 1907), Alzheimer’s disease (AD) has become the most prevalent form of dementia, accounting for 60 to 80 percent of cases. The World Health Organization has recognized AD as a key disease for global public health. It currently affects approximately 47.5 million people (Vos et al., 2017), predominantly those aged 65 years and older. The latest epidemiological data shows that there are currently approximately 44 million people with dementia worldwide. Due to the accelerating global ageing trend, the number of dementia patients is expected to continue increasing, doubling approximately every 5 years (D'Cruz and Banerjee, 2021), and potentially reaching 152 million by 2050. This upward trend exhibits marked regional disparities, with the most substantial increases expected in low- and middle-income countries (Patterson, 2018). When adopting the biological definition of AD, the actual prevalence may be up to three times higher than that based on clinical diagnosis, thereby further compounding the associated social and economic burden (Prince et al., 2014; Ganguli et al., 2005).

Despite over a century of research since its discovery, the underlying pathogenesis of AD remains poorly understood. Although some treatments can temporarily alleviate symptoms (Yiannopoulou and Papageorgiou, 2020; Livingston et al., 2017), no curative therapy is currently available. The pathological hallmarks of AD include extracellular amyloid-β (Aβ) plaques (Haass and Selkoe, 2007) and intracellular neurofibrillary tangles (NFTs) (Guan et al., 2021; Sebastián-Serrano et al., 2018). Additionally, neuroinflammation (Leng and Edison, 2021), oxidative stress (Bai et al., 2022), cholinergic dysfunction (Francis et al., 1999), genetic predispositions (Latimer et al., 2021), mitochondrial impairment (Song et al., 2021), gut microbiota dysbiosis (Cryan et al., 2019; Angelucci et al., 2019), and compromised blood–brain barrier (BBB) integrity (Sweeney et al., 2018) have also been implicated in the neurodegenerative processes of AD. Against this backdrop, elucidating the pathological mechanisms of AD and developing effective therapeutic strategies have become urgent priorities in geriatric research.

In recent years, significant progress has been made in the investigation of natural compounds for the treatment of AD. A plethora of studies have reported the potential therapeutic effects of individual herbal medicines or their extracts, such as baicalein (Siddiqui et al., 2024a), punicalagin (Siddiqui et al., 2024b), ginsenosides (She et al., 2024), quercetin (Khan et al., 2019), salidroside (Zhang N. et al., 2023), naringin (Singh and Kumar Singh, 2024), and astragalosides (Ding et al., 2022), in AD management. These studies primarily focus on the regulation of multiple signaling pathways by natural compounds, including PI3K/Akt (Fakhri et al., 2021; Long et al., 2021), autophagy (Zhang Z. et al., 2021), Nrf2 (George et al., 2022), the cholinergic system (Hampel et al., 2018), the gut–brain axis (Zhang T. et al., 2023), glutamate signaling (Puranik and Song, 2024), and STAT3 (Wen and Hu, 2024). These compounds interfere with the core pathological mechanisms of AD, thereby demonstrating multi-target and integrative therapeutic potential. However, despite the critical role of the MAPK inflammatory signaling pathway in the pathogenesis and progression of AD, systematic reviews addressing its regulation by natural compounds remain scarce. Therefore, a comprehensive summary of the mechanisms by which natural compounds modulate the MAPK pathway in the treatment of AD is of substantial research significance.

## 2 Neuroinflammation and AD

Neuroinflammation, a significant mechanism underlying NDDs, has become a major focus of AD research in recent years. In AD, neuroinflammation plays a critical role in disease initiation, pathological progression, and clinical deterioration (Kinney et al., 2018; Kwon and Koh, 2020; Newcombe et al., 2018), primarily characterized by excessive activation of microglia and astrocytes, along with the involvement of multiple pro-inflammatory mediators (Uddin et al., 2020). In the early stages of AD, glial cells recognize pathological Aβ and tau proteins via pattern recognition receptors (e.g., TLRs, TREM2), which then mediate their clearance and exert neuroprotective effects (Temviriyanukul et al., 2023; Liu J. et al., 2020). However, as the disease progresses, sustained abnormal glial activation triggers the overactivation of signaling pathways such as NF-κB and p38 MAPK, leading to the excessive release of pro-inflammatory cytokines (e.g., IL-1β, TNF-α, IL-6) (Newcombe et al., 2018; Liao et al., 2021) and reactive oxygen/nitrogen species (ROS/RNS) (Temviriyanukul et al., 2023), thereby establishing a chronic neuroinflammatory microenvironment (Walters et al., 2016).

This persistent inflammatory state interacts with Aβ deposition and tau hyperphosphorylation, forming a vicious cycle. On one hand, inflammatory mediators promote the pathological changes in tau protein by activating kinases such as GSK-3β (Laurent et al., 2018). Furthermore, aggravation of these pathological changes in tau protein has been demonstrated to further amplify glial activation (Leng and Edison, 2021; Song et al., 2021; Laurent et al., 2018; Hickman et al., 2008). Notably, neuroinflammation exhibits dual regulatory roles: acute inflammation has a certain neuroprotective effect, while chronic inflammation can accelerate the decline of cognitive function by inducing synaptic damage and neuronal death.

The process is influenced by a variety of endogenous and exogenous factors. The endogenous factors encompass sex (e.g., estrogen deficiency) (Moser and Pike, 2016; Maioli et al., 2021; Cui et al., 2013; Lee et al., 2014; Chakrabarti et al., 2014; Tecalco-Cruz et al., 2021; Yun et al., 2018), aging (Hoozemans et al., 2011), and genetic mutations such as TREM2 R47H (Fuller et al., 2010) and ApoE4 (Kloske and Wilcock, 2020). In addition, exogenous factors include chronic stress-induced activation of the hypothalamic–pituitary–adrenal (HPA) axis (Justice, 2018; Lesuis et al., 2018; Carroll et al., 2011), heavy metal exposure (Teleanu et al., 2022; Bondy, 2021; Harischandra et al., 2019), metabolic disorders (e.g., obesity and diabetes) (Nuzzo et al., 2015), and Western diet-induced gut microbiota dysbiosis (Cavaliere et al., 2019; McGrattan et al., 2019; Chen et al., 2022; Wang X. et al., 2019; Colombo et al., 2021; Lin et al., 2022; Vogt et al., 2017). Gut dysbiosis has been demonstrated to promote central neuroinflammation and Aβ accumulation through mechanisms such as abnormal short-chain fatty acid metabolism, inflammatory signaling activation, and disruption of BBB integrity.

In summary, neuroinflammation serves not only as a key bridge between the two pathological mechanisms of Aβ and tau, but also as an important target for the early diagnosis and therapeutic intervention of AD.

## 3 MAPK signaling pathway and AD

Microglia and astrocytes in the central nervous system (CNS) are activated via multiple molecular signaling pathways, leading to the release of various inflammatory mediators, including nuclear factor-κB (NF-κB), p38 MAPK, mammalian target of rapamycin (mTOR), cyclooxygenase (COX), peroxisome proliferator-activated receptor-γ (PPAR-γ), and the NLRP3 inflammasome. This activation process has been shown to trigger neuronal and synaptic damage as well as neuronal apoptosis, thereby accelerating the pathological progression of AD.

The MAPK signaling pathway family plays a pivotal regulatory role in AD pathogenesis. The three primary subpathways are all significantly activated in the damaged neurons of AD patients, which are named extracellular signal-regulated kinase (ERK), c-Jun N-terminal kinase (JNK), and p38 MAPK. This activation indicates the involvement of the MAPK pathway in the pathophysiological processes and pathogenesis of AD. The MAPK pathway is extensively implicated in key pathological processes of AD, including neuroinflammation, tau hyperphosphorylation, synaptic dysfunction, neuronal apoptosis, and oxidative stress.

ERK is predominantly activated by growth factors, which play critical roles in cell differentiation, proliferation, and development. Conversely, JNK and p38 MAPK are known to be activated by mitogens, cytokines, cell death receptors, and various stress stimuli, including oxidative stress, heat shock, hypoxia, and ultraviolet radiation.

The pathogenesis of AD is intricate, and in recent years, the p38 MAPK signaling pathway has emerged as a research hotspot (Jia et al., 2012). p38 MAPK, a key protein that is abundantly expressed in multiple brain regions associated with cognitive function, can be activated by various inflammatory mediators, including cytokines, chemokines, and bacterial lipopolysaccharides (LPS).

In the process of glial cell-mediated neuroinflammation, activated microglia generate substantial amounts of neurotoxic mediators, including IL-1β, TNF-α, COX-2, and inducible nitric oxide synthase (iNOS), via the p38 MAPK signaling pathway. These inflammatory factors further activate the p38 MAPK pathway in astrocytes, thereby promoting the formation of an inflammatory cycle that is difficult to halt.

Mechanistic studies suggest that p38 MAPK exerts a detrimental effect on neurons by inducing abnormal tau phosphorylation, mitochondrial dysfunction, and apoptosis but also disrupts glutamate homeostasis and synaptic plasticity through activation of the NF-κB signaling pathway (Singh, 2022). Furthermore, evidence suggests a direct correlation between aberrant microglia activation during the initial stages of AD and the subsequent development of synaptic dysfunction and neuronal death (Leng and Edison, 2021).

Clinical studies have demonstrated that p38 MAPK activity in the brain tissue of AD patients is significantly elevated compared to healthy controls (Lee and Kim, 2017; Kheiri et al., 2018). These findings provide a critical theoretical basis for targeted regulation of the MAPK pathway in the treatment of AD.

Research has demonstrated that the inhibition of Aβ toxicity and tau protein hyperphosphorylation, with the objective of protecting neurons, as well as the reduction of neuroinflammation by inhibiting the p38 MAPK pathway, are the key mechanisms by which this signaling pathway exerts therapeutic effects. Excessive Aβ deposition has been demonstrated to result in neuronal damage, induce a cytotoxic reaction, activates inflammatory signaling pathways, induces neuroinflammation and oxidative stress responses, and simultaneously damages the long-term enhancement (LTP) function of the synapses in the hippocampal region. Furthermore, excessive deposition of Aβ induces cellular stress, leading to the occurrence of neuroinflammation, which stimulates astrocytes to release inflammatory cytokines (e.g., TNF-α, IL-1β), thereby activating the p38 MAPK signaling pathway. Consequently, the activity of its downstream nuclear factor -κB (NF-κB) also increases accordingly, further promoting the release of pro-inflammatory factors and thereby exacerbating the neuroinflammatory response.

In addition, the inhibition of this pathway has been shown to reduce levels of reactive oxygen species (ROS) and superoxide (O_2_^−^), and downregulates the expression of nsy-1, sek-1, and pmk-1 mRNA (Li et al., 2018). This, in turn, has been demonstrated to mitigate oxidative stress and reduce Aβ plaque formation, ultimately exerting anti-AD effects. Another hallmark of AD pathology is the formation of neurofibrillary tangles, primarily composed of hyperphosphorylated tau protein. Under normal conditions, tau proteins are predominantly localized in neuronal axons, and participate in maintaining the stability of microtubules. The process of hyperphosphorylation of tau results in the impairment of its microtubule-binding capacity. This, in turn, leads to the destabilization of the cytoskeleton and the disruption of axonal transport. These phenomena contribute to the manifestation of synaptic dysfunction.

**FIGURE 1:**
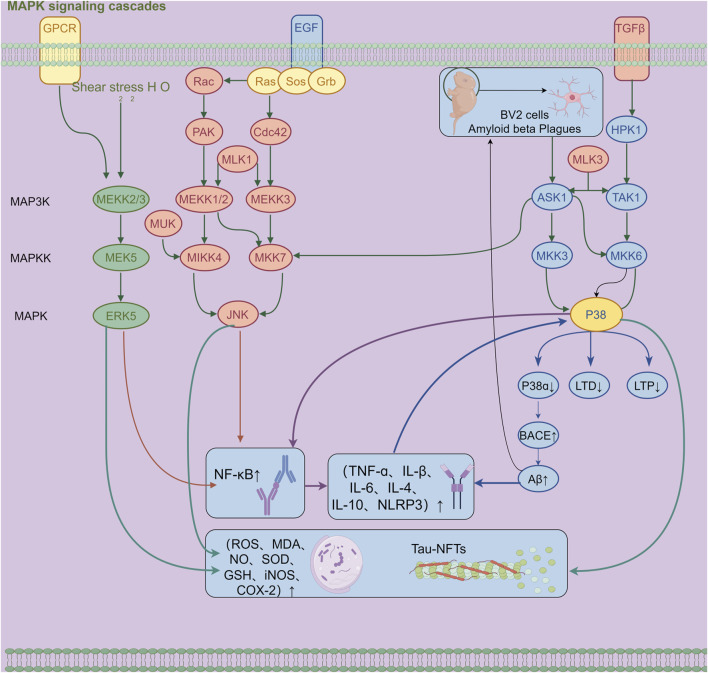
Mechanism diagram of β-amyloid-induced activation of the MAPK signaling pathway and neuroinflammatory response in BV2 microglia (300 dpi.Thanks for Figdraw).

Synapses serve as the fundamental structures that regulate neural functions and directly participate in the transmission of neural signals. Among them, LTP plays a key role in the formation of learning and memory and is an important physiological basis for both. Studies have revealed that activation of p38 MAPK can inhibit LTP and reduce synaptic plasticity in the hippocampus, thereby directly affecting the process of memory formation. Therefore, inhibiting the activation of the p38 MAPK signaling pathway helps improve synaptic dysfunction and restore synaptic plasticity, which is a potentially effective strategy for intervening in AD (Yu et al., 2018; Figure 1).

## 4 Traditional Chinese medicine (TCM) compounds treat AD through the MAPK signaling pathway

A substantial body of research has demonstrated that plant-derived natural compounds—such as flavonoids, alkaloids, saponins, and phenolic acids—modulate neurotransmitter levels through multiple signaling pathways and cascade reactions, thereby improving behavioral performance and memory function, reducing Aβ protein deposition, inhibiting acetylcholinesterase (AChE) activity, preventing neuronal apoptosis, and enhancing cerebral antioxidant capacity. Moreover, accumulating evidence indicates that these compounds can specifically modulate the MAPK signaling pathway, demonstrating considerable therapeutic potential in the treatment of AD (Table 1, 2).

**TABLE 1:** Plant-derived natural compounds modulating the MAPK signaling pathway in clinical studies for the prevention and treatment of Alzheimer’s disease.

Class	Active ingredients	Study subjects	Impact on	Refs
Flavonoids	Myricetin (ME)	3×Tg-AD triple transgenic mice Aβ25-35 was used to induce BV2 cells	↓: IL-1β, TNF-α, IL-6 ↑: IL-4, IL-10, NLRP3, ASC, caspase-1, IL-18, TFAM, NRF1	Liu et al. (2023)
		Chinese hamster lung fibroblasts (V79-4) cells	↑: Bcl-2, Akt ↓: Bax, p38 MAPK, JNK	Ramezani et al. (2016), Wang et al. (2017), Chen et al. (1997)
	Nobiletin (NOB)	C57BL/6J mice	↑: (Vamp1), Snap-25, Psd-95 ↓: iNOS, COX-2, TLR-4, IL-1β, TNF-α, IL-6, IL-1β, TNF-α mRNA, ROS, H2O2, AKT, JNK, ERK, p38	Qi et al. (2019)
Terpenoids	Paeoniflorin (Pae)	Transgenic mice	↑: Bcl-2/Bax, p-Akt ↓: NF-κB p65, TNF-α, IL-1β, IL-6, Caspase-3, p-p38 MAPK	Gu et al. (2016)
	Ganoderic Acid A (GAA)	HT22 cells	↑: SOD, T-AOC ↓: p-ERK, p-JNK, p-p38, MDA, ROS, caspase-3, p-Tau, Aβ	Shao et al. (2025)
	Huperzine A (Hup A)	SHSY5Y neuroblastoma cells	↑: NGF, P75NTR, TrkA mRNA, MAP/ERK	Tang et al. (2005)
	Triptolide (TP)	APP/PS1 mice	↓: MAPK, p38, ERK, JNK	Cui et al. (2016)
		HT22 cells	↓: MKP-1, siRNA, MAPKs, ERK-1/2, p38 MAPK, JNK-1/2	Koo et al. (2009)
Phenols	Gallic acid (GA)	C57BL/6 mice, HUVEC, PC12, SH-SY5Y, HT22	↑: GSH, CAT ↓: ROS, Ca2+, Gadd45b, Gadd45g, p38/MAPK	Wan et al. (2025)
	Resveratrol (RSV)	Male mice aged from 7 to 9 weeks	↑: SIRT1 ↓: P-p38 MAPK	Zhao et al. (2022)
Other classes	P. Ginseng (BGE)	5xFAD mice	↓: TNF-α, IL-6, COX-2, iNOS, p38 MAPK, NF-κB, STAT3, NLRP3, Nrf2, HO-1, TLR-2, TLR-4	Ha et al. (2025)
		WT; C57BL/6J mice5xFAD mice	↑: Nrf2, HO-1 ↓: Aβ, p-tau, IL-6, TNF-α, COX-2, iNOS, p-p38 MAPK, p-NF-κB p65, p-STAT3, NLRP4, NLRP3, ASC, IL-1β, caspase-1, TLR2, TLR4	Ha et al. (2025)
	Schisandrin a (SCH A)	LPS-induced inflammation and oxidative stress in RAW 264.7 macrophages	↑: Nrf2, HO-1 ↓: Keap1, NO, PGE2, TNF-α, IL-1β, iNOS, COX-2 mRNA, IκB-α, NF-κB p65(JNK), p38 MAPK, ERK, PI3K, Akt, ROS	Kwon et al. (2018)
		SH-SY5Y and SK-N-SH cells	↑: SOD, p-ERK1/2, ERK1/2, GSH ↓: MDA, ROS, IL-6, IL-1, TNF	Jia et al. (2024)
	Crocin	SH-SY5Y PC12 cell	↑: GSK3β ↓: BACE, APP-C99, tau, pThr231, pSer199/Ser202, GSK3β, ERK2, pERK1, pERK2	Chalatsa et al. (2019)
	Ginsenosides (Re, Rg1, Rg2)	Male C57BL/6 mice	↓: TNF-α, NO, iNOS, IκB, NFκB, p38, ERK1/2, JNK	Hu et al. (2011)
		LPS-induced BV2 cell	↓: iNOS, COX-2, TNF-α, IL-1β, NF-κB, IκB-α, CREB, ERK1/2, JNK, p38 MAPK	Zong et al. (2012)
		Adult female Wistar rats LPS	↑: GR ↓: TNF-α, IL-1β, IκB-α, NFκB, ERK1/2, JNK, p38 MAPK	Sun et al. (2016)
		Male 7-month-old B6-Tg (APPSwe, tauP301L) Ps1tm1 (3xTg-AD) mice	↑: CD31, p-ERK/ERK, p-MAPK/MAPK ↓: Aβ25-35, TNF-α, IL-1β, IL-6, GFAP, ICAM-1, VCAM-1 mRNA, Aβ, p-Tau/Tau	Ye et al. (2023)
	Sesame oil (SO)	BV-2 cell	↓: iNOS mRNA, p38 MAPK, NO, ROS	Hou et al. (2003)
		RAW 264.7 cell	↑: Nrf2, HO-1, AMPK ↓: E2 (PGE2), NO, iNOS, COX-2, NF-κB, MAPK	Wu et al. (2015)
		AlCl3-induced AD mice	↑: BDNF, PPAR-γ ↓: NF-κB, p38MAPK, Aβ, TNF-α, IL-1β, AChE	Mohamed et al. (2021)
	1,6-O, O-diacetylbritannilactone (OABL)	5xFAD mice	↑: GSH ↓: NO, PGE2, TNF-α, iNOS, COX-2, NF-κB, MDA, T-SOD	Tang et al. (2022)
	Esculin (ESC)	C57BL/6J 6	↑: p-ERK 1/2 ↓: TNF-α, IL-6, SOD, MDA, MCP-1, ICAM-1, AP-1, p-p38 MAPK, p-JNK	Song et al. (2018)
	Berberine (BBR)	MaleC57BL/6J mice	↑: ChAT, GSH-PX, GSH, SOD ↓: AchE, MMP-3/9, MDA, TNF-α, IL-6, caspase-3, Bax, p38 MAPK	Wang and Zhang (2018)
		microglial and BV2 cell	↓: IL-6, MCP-1, COX-2, iNOS, NF-κB p65, Akt, p38, ERK1/2	Jia et al. (2012)

**TABLE 2:** Sources, Bioactivity, and chemical structures of plant-derived natural compounds.

Class	Active ingredients	Source	Biological activities	Structures	Refs
Flavonoids	Myricetin (ME)	Myricaceae and Euphorbiaceae	anti-inflammatory activity antioxidant properties, improvement of mitochondrial dysfunction, and regulation of autophagy	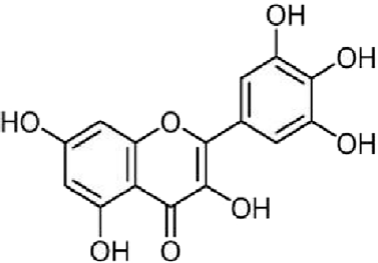	Taheri et al. (2020)
	Nobiletin (NOB)	Citrus fruits of the Rutaceae family	anti-inflammatory, antioxidant, antiatherosclerotic, neuroprotective, and anti-obesity effects	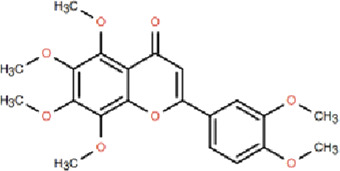	Chen et al. (1997), Nogata et al. (2006), Tanaka et al. (2004), Choi et al. (2007), Hirata et al. (2008), Cui et al. (2010), Lee et al. (2010), Yoshim et al. (2004), Miyamoto et al. (2008), Lam et al. (2011), Lee et al. (2011), Mulvihill et al. (2011), Choi et al. (2011), Kanda et al. (2012)
Terpenoids	Paeoniflorin (Pae)	Paeonia lactiflora pall	anti-inflammatory, antioxidant, antithrombotic, antidepressant	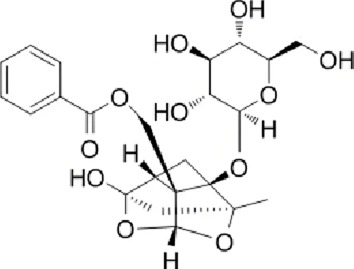	Chen et al. (2011), Ye et al. (2016), Hino et al. (2012), Qiu et al. (2013), Zhang et al. (2009)
	Ganoderic Acid A (GAA)	Ganoderma lucidum	anti-inflammatory, antioxidant, neuropsychopharmacological	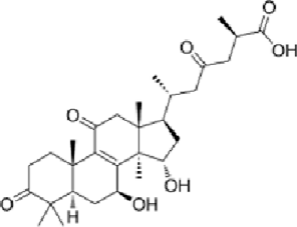	Jiang et al. (2018), Meng et al. (2020), Wan et al. (2019), Lixin et al. (2019), Yang et al. (2018), Zhang et al. (2020), Zhang et al. (2021b), Zheng et al. (2022)
	Huperzine A (Hup A)	Huperzia Serrata	modification of β-amyloid peptide processing, reduction of oxidative stress, neuronal protection against apoptosis, and regulation of the expression and secretion of nerve growth factor (NGF) and NGF signaling	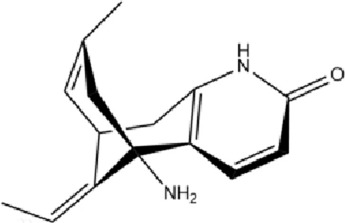	College (1985)
	Triptolide (TP)	Celastraceae	anti-inflammatory, immunomodulatory, and anti-aging effects	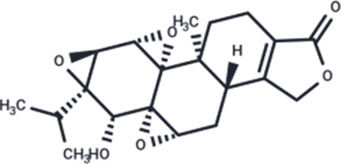	(Chen et al., 2018), (Gao et al., 2021; Tong et al., 2021)
Phenols	Gallic acid (GA)	Tea leaves, oranges, papayas, pomegranates, and cardamom	antioxidant effects	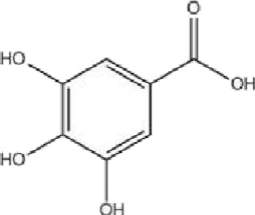	Latha and Daisy (2011), Gu et al. (2025)
	Resveratrol (RSV)	Grapes	anti-angiogenic, immunomodulatory, neuroprotective and cardiovascular disease (CVD) preventive effects	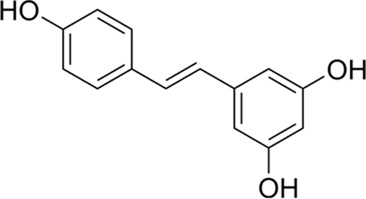	Vestergaard and Ingmer (2019), Breuss et al. (2019)
Other classes	P. Ginseng (BGE)	Panax ginseng	immunomodulation, antioxidant effects, anti-fatigue properties, and cardiovascular protection	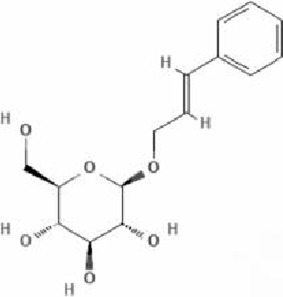	Metwaly et al. (2019)
	Schisandrin a (SCH A)	Schisandra chinensis	anti-inflammatory, antioxidant, neuroprotective	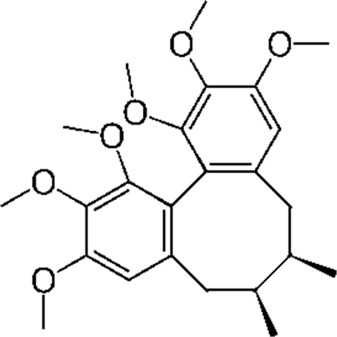	Choi, 2018; Cui et al. (2020), Kong et al. (2018), Meng et al. (2019), Jeong et al. (2019), Wang et al. (2014), Zhang et al. (2010)
	Crocin (CRO)	Crocus sativus stigmas、Gardenia jasminoides Ellis	antioxidant activities	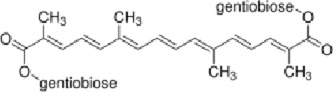	Liu et al. (2020b), Khorasany and Hosseinzadeh (2016)
	Ginsenosides (Re, Rg1, Rg2)	Ginseng	enhancing cognitive function, inhibiting apoptosis, and exerting neuroprotective activities	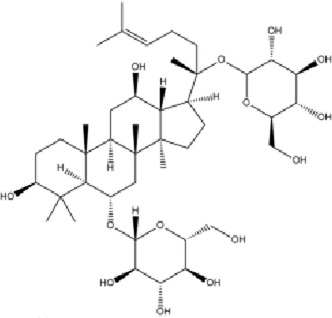	Li et al. (2020)
	Sesame oil (SO)	Sesame	anti-inflammatory, antioxidant, and cardiovascular protective effects	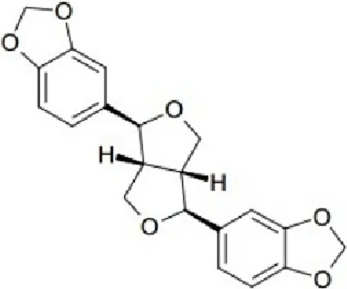	Jayaraj et al. (2020)
	1,6-O, O-diacetylbritannilactone (OABL)	Impatiens grandiflorum	anti-inflammatory and neuroprotective effects	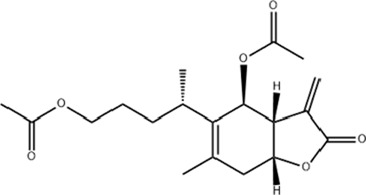	Shi et al. (2022), Zhao et al. (2006)
	Berberine (BBR)	*Berberis vulgaris*	anti-inflammatory, cardioprotective, neuroprotective	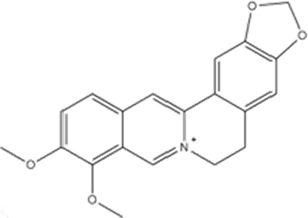	(Ye et al., 2009), (Ma et al., 1999; Küpeli et al., 2002; Le Tran et al., 2003; Zheng et al., 2003; Kettmann et al., 2004; Račková et al., 2004; Letašiová et al., 2006)
	Esculetin (ESC)	Rutaceae and Umbelliferae and essential oils of cinnamon bark, cassia leaf, and lavender oil	antioxidant, antiinflammatory, antidiabetic, neuroprotective	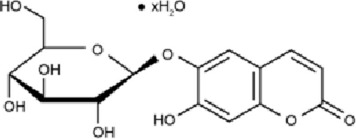	Bhattarai et al. (2021), Orioli et al. (2024), Pisani et al. (2022)

### 4.1 Flavonoids

#### 4.1.1 Myricetin (ME)

ME is a naturally occurring flavonoid with diverse biological activities. It is predominantly found in plant species belonging to the Myricaceae and Euphorbiaceae families, and the main sources include plant extracts such as berries and tea leaves (Taheri et al., 2020). Studies have demonstrated that ME exerts a broad range of pharmacological effects, including anti-inflammatory activity (by modulating the NLRP3 inflammasome, the NF-κB pathway, and various pro-inflammatory cytokines), antioxidant properties, improvement of mitochondrial dysfunction, and regulation of autophagy.

Liu. et al. (Liu et al., 2023) employed the 3×Tg-AD mouse model and integrated network pharmacology with molecular docking analysis to predict that ME can regulate the MAPK signaling pathway through multiple targets. The experimental findings revealed that ME significantly inhibited activation of the p38 MAPK pathway, thereby alleviating Aβ25-35-induced mitochondrial dysfunction, suppressing overactivation of the NLRP3 inflammasome, and improving cognitive and memory deficits in AD model mice.

Furthermore, Kyoung et al. (Kang et al., 2010; Ramezani et al., 2016; Wang et al., 2017) also found that ME has a neuroprotective effect on oxidative stress-induced mitochondrial-dependent and caspase-dependent apoptosis processes by regulating the p38 MAPK and JNK signaling pathways, suggesting that it may exert an anti-AD effect by regulating the MAPK signaling pathway.

#### 4.1.2 Nobiletin (NOB)

NOB is a naturally occurring flavonoid that is predominantly found in the peel of citrus fruits belonging to the Rutaceae family (Chen et al., 1997; Nogata et al., 2006). Studies have demonstrated that NOB exhibits multiple pharmacological activities, including anti-inflammatory, antioxidant, anticancer, antidiabetic, antiatherosclerotic, neuroprotective, and anti-obesity effects (Tanaka et al., 2004; Choi et al., 2007; Hirata et al., 2008; Cui et al., 2010; Lee et al., 2010; Yoshim et al., 2004; Miyamoto et al., 2008; Lam et al., 2011; Lee et al., 2011; Mulvihill et al., 2011; Choi et al., 2011; Kanda et al., 2012). According to extant research, NOB has been demonstrated to ameliorate AD-related cognitive impairments, such as decline in learning and memory, by inhibiting apoptosis, alleviating oxidative stress, and reducing cerebral Aβ protein levels.

Neuroinflammation has been identified as a significant mechanism contributing to the progression of NDDs. In the mouse microglial BV-2 cell line, NOB exhibits potent anti-neuroinflammatory effects, significantly inhibiting the production and release of LPS-induced pro-inflammatory mediators, including NO, TNF-α, IL-1β, and IL-6 (Cui et al., 2010; Ho and Kuo, 2014; Wang Y. et al., 2019). A recent study demonstrated that mice administered 100 mg/kg of nobiletin daily for 6 weeks exhibited effective alleviation of LPS-induced memory impairment. The study also found that nobiletin inhibited the activation of microglia and the secretion of related pro-inflammatory cytokines.

Qi et al. (2019) further demonstrated that NOB treatment significantly decreased serum levels of iNOS, COX-2, TLR4, IL-1β, and TNF-α, inhibited NF-κB nuclear translocation, and enhanced phosphorylation and activation of key signaling proteins including AKT, JNK, ERK, and p38 MAPK.

Further experiments demonstrated that under NOB treatment, inhibitors of ERK (U0126), p38 (SB203580), and JNK (SP600125) can synergistically alleviate the inflammatory response induced by LPS, confirming that NOB may alleviate the inflammatory state of BV-2 microglia by regulating the MAPK signaling pathway.

In summary, NOB effectively alleviates inflammation-induced cognitive deficits and neuroinflammation by reducing neuronal damage, inhibiting microglial activation, suppressing inflammatory factor release, and restoring mitochondrial function, thereby highlighting its potential therapeutic value in the prevention and treatment of AD.

### 4.2 Phenols

#### 4.2.1 Gallic acid (GA)

GA is a polyphenolic organic compound that is found in plants such as tea leaves, oranges, papayas, pomegranates, and cardamom (Latha and Daisy, 2011). It has been demonstrated that GA exhibits a variety of pharmacological activities, including anticancer and antioxidant effects, and has been extensively applied in medical research (Gu et al., 2025). Studies have demonstrated that GA reduces brain injury by decreasing infarct size in rat models of cerebral ischemia (Kumar et al., 2021). In neurological disorder models, GA significantly ameliorates cognitive impairment in rotenone-induced Parkinson’s disease (PD) rat models (Sheikhpour et al., 2023). Related studies indicate that GA improves learning and memory performance and enhances motor function in AD mice. As demonstrated in the experimental data from Wan et al. (2025), the application of GA has been shown to attenuate the damage induced by glutamate (Glu) in PC12, SH-SY5Y, and HT22 neural cells, exhibiting a dose- and time-dependent response. The protective effects of GA are more pronounced in PC12 and SH-SY5Y cells. Mechanistic studies suggest that GA mitigates AD pathological progression by inhibiting the expression of Gadd45g and Gadd45b and their downstream P38/MAPK signaling pathway, thereby attenuating oxidative stress responses. Transcriptomic analyses further reveal that the P38/MAPK signaling pathway plays a critical role in mediating GA’s neuroprotective effects against AD. In summary, GA attenuates the progression of AD by inhibiting the P38/MAPK signaling pathway. It has potential value as a natural candidate drug for the prevention of AD.

#### 4.2.2 Resveratrol (RSV)

Resveratrol, a naturally occurring phenolic compound, functions as a plant antitoxin. It is produced in response to mechanical damage or attack by pathogens, including bacteria and fungi (Vestergaard and Ingmer, 2019). It exhibits diverse pharmacological activities, including anti-angiogenic, immunomodulatory, antibacterial, neuroprotective, anticancer, antidiabetic, and cardiovascular disease (CVD) preventive effects (Breuss et al., 2019). Resveratrol has been demonstrated to effectively ameliorate mitochondrial dysfunction, mitigate oxidative stress, modulate inflammatory responses, and inhibit apoptosis. Moreover, preliminary studies suggest that resveratrol may also have an improving effect on NDDs (Fantacuzzi et al., 2022). In aged C57BL/6 mice, oral administration of resveratrol at 200 mg/kg for 10 consecutive days restored brain microvascular endothelial function and suppressed ROS production, thereby improving the coupling response of cortical neurovascular and promoting neuronal activity and functional recovery (Toth et al., 2014). In a C57BL/6 mouse model, intraperitoneal injection of 100 mg/kg resveratrol for seven consecutive days alleviated hippocampus-dependent cognitive deficits via anti-inflammatory and anti-apoptotic mechanisms (Li et al., 2014). In male F344 rats, intraperitoneal administration of resveratrol at 40 mg/kg for 4 weeks significantly improved memory and emotional functions, facilitated hippocampal neurogenesis and microvascular remodeling, and suppressed glial cell activation (Kodali et al., 2015). Zhao et al. (2022) demonstrated that resveratrol ameliorates post-traumatic cognitive dysfunction in mice by activating the deacetylase Sirtuin one and inhibiting phosphorylation of p38 MAPK. Studies suggest that p38 MAPK is activated following traumatic brain injury (TBI), and resveratrol exerts regulatory effects on this pathway.

### 4.3 Terpene

#### 4.3.1 Paeoniflorin (Pae)

Pae is a water-soluble monoterpene glucoside predominantly extracted from the dried roots of Paeonia lactiflora Pall., a species within the Paeoniaceae family. It constitutes the major active component of total paeony glycosides (TGP), comprising over 40% of the total glycoside content. Pae exhibits diverse pharmacological effects, such as anti-inflammatory, antioxidant, antithrombotic, anticonvulsant, antidepressant, sedative, analgesic, antispasmodic, and immunomodulatory activities (Chen et al., 2011; Ye et al., 2016; Hino et al., 2012; Qiu et al., 2013; Zhang et al., 2009). Pae modulates multiple signaling pathways, including G protein-coupled receptors (GPCRs), MAPKs/NF-κB, PI3K/Akt/mTOR, JAK2/STAT3, and TGF-β/Smads pathways. Pae has been shown to regulate calcium ion (Ca^2+^) and reactive oxygen species (ROS) homeostasis, thereby exerting therapeutic effects against NDDs.

Gu et al. (2016) established an AD model using transgenic mice and demonstrated that Pae exerts significant neuroprotective effects, markedly improving cognitive functions in AD mice, as evidenced by enhanced escape distance and latency performance. The study revealed that Pae inhibits apoptosis by elevating the Bcl-2/Bax ratio and p-Akt expression in brain tissue of AD mice, concurrently downregulating p-P38 MAPK expression. This results in the attenuation of inflammatory responses and caspase-3 activity. Further investigations suggest that prolonged Pae treatment suppresses JNK and P38 MAPK activation while enhancing ERK activation. Pae effectively reverses ischemia-induced activation of the NF-κB signaling pathway and exerts marked neuroprotective effects in rats with cerebral ischemic injury by mitigating inflammatory responses within brain tissue.

#### 4.3.2 Ganoderic acid a (GAA)

GAA is a triterpenoid compound isolated from Ganoderma lucidum (reishi mushroom) has been shown to possess inherent natural neuroprotective properties. GAA exhibits diverse pharmacological activities, including anti-inflammatory, antioxidant, antitumor, neuropsychopharmacological, hepatoprotective, cardioprotective, nephroprotective, and pulmonary protective effects by modulating various signal transduction pathways (Jiang et al., 2018; Meng et al., 2020; Wan et al., 2019; Lixin et al., 2019; Yang et al., 2018; Zhang et al., 2020; Zhang L. et al., 2021; Zheng et al., 2022), underscoring its substantial clinical application potential. In a *Caenorhabditis elegans* model, GAA treatment significantly delayed cellular senescence and extended healthspan (Chen et al., 2025). Studies have reported that in an Aβ42-induced AD mouse model, GAA activates the Axl receptor tyrosine kinase (Axl)/CDC42-associated kinase 1 (Pak1) signaling pathway, stimulates autophagy in BV2 microglial cells, enhances Aβ42 clearance, and subsequently ameliorates cognitive deficits (Qi et al., 2021). Furthermore, GAA dose-dependently increased the viability of HT22 cells injured by Aβ25-35, while concurrently suppressing the expression of MAPK pathway-related proteins. GAA markedly downregulates cleaved caspase-3 levels, decreases apoptosis, and suppresses Aβ and phosphorylated tau (p-Tau) expression via inhibition of the ERK signaling pathway (Shao et al., 2025). Given its ability to inhibit apoptosis via the ERK/MAPK signaling pathway, GAA shows broad prospects as a potential candidate drug for the treatment of AD.

#### 4.3.3 Huperzine A (Hup A)

Elsholtzia ciliata, more commonly referred to as the Thousand-layer Tower, is a traditional Chinese medicinal herb that belongs to the Huperziaceae family. Modern studies have identified alkaloids and triterpenoids as the main bioactive constituents, among which Hup A is the important active compound. Hup A has been successfully utilized in the treatment of AD, dementia, and myasthenia gravis (College, 1985). It has been demonstrated that this agent functions by impeding the phosphorylation of p38 MAPK and ERK within the MAPK signaling pathway, leading to decreased expression of iNOS and cyclooxygenase-2 (COX-2), thereby suppressing the release of pro-inflammatory mediators. Hup A exhibits neurotrophic effects against oxidative stress by promoting nerve growth factor (NGF) synthesis in SH-SY5Y cells, a process contingent upon activation of the MAPK/ERK signaling pathway (Tang et al., 2005). Furthermore, the MAPK/ERK signaling pathway has been implicated in mediating the neuroprotective effects of Hup A in transient cerebral ischemia-reperfusion animal models (Wang et al., 2006). The MAPK/ERK signaling pathway plays a pivotal role in regulating various biological processes, including proliferation, differentiation, and the expression of multiple transcription factors.

#### 4.3.4 Triptolide (TP)

The genus Tripterygium, which belongs to the family Celastraceae, contains Tripterygium lactone, a natural diterpenoid compound that is one of its principal bioactive constituents (Chen et al., 2018). This compound has been demonstrated to possess a wide range of pharmacological activities, including anti-inflammatory, immunomodulatory, antitumor, and anti-aging effects (Gao et al., 2021; Tong et al., 2021). Tripterygium glycoside has been identified as a regulator of β-amyloid (Aβ) levels, with the capacity to mitigate synaptic dysfunction and memory impairments associated with AD. Owing to its high lipophilicity and low molecular weight, Tripterygium glycoside has been observed to cross the blood-brain barrier (BBB), thereby demonstrating potential therapeutic efficacy in treating neurological disorders (Zhang et al., 2012). Reduction of oxidative stress is regarded as a key protective mechanism of Tripterygium wilfordii heterophyllum against AD; however, its potential preventive effects on AD pathology via anti-inflammatory pathways require further elucidation. TP inhibits the expression of MKP-1, which primarily deactivates ERK1/2, p38 MAPK, and JNK1/2 signaling pathways, thereby exerting anti-proliferative and pro-apoptotic effects (Koo et al., 2009). Cui et al. (2016) demonstrated that Tripterygium wilfordii significantly suppresses microglial activation in the cerebral cortex and hippocampus of APP/PS1 transgenic mice. Recent molecular biology studies have identified the MAPK signaling pathway is one of the core mechanisms for regulating inflammatory responses. Treatment with Tripterygium wilfordii lactone markedly reduced phosphorylation levels of p38, ERK, and JNK in the brain tissue of APP/PS1 mice, indicating inhibition of MAPK pathway activation. Furthermore, Tripterygium wilfordii lactone has been shown to suppress the expression of pro-inflammatory cytokines TNF-α and IL-1β, effects that are likely linked to its inhibitory action on the MAPK signaling pathway.

### 4.4 Other categories

#### 4.4.1 1,6-O, O-diacetylbritannilactone (OABL)

OABL is a natural 1,10-bislactone-type sesquiterpene lactone compound that has been isolated from Impatiens grandiflorum (Shi et al., 2022). It exhibits a broad spectrum of pharmacological activities and has been applied in the treatment of bronchitis, diabetes, intestinal ulcers, gastrointestinal disorders, and various inflammatory conditions (Zhao et al., 2006). In addition, OABL has demonstrated encouraging efficacy in the treatment of AD. Its anti-inflammatory mechanisms mainly involve the inhibition of inflammatory mediator production (including NO, PGE2, TNF-α, iNOS, and COX-2) and the suppression of nuclear translocation of the transcription factor NF-κB (Chen et al., 2017). Furthermore, OABL has been shown to possess antioxidant properties that protect neurons against oxidative damage (Wang et al., 2022). In AD animal models, OABL has been shown to significantly improve cognitive performance, restore neuronal morphology in the hippocampus, reduce Aβ amyloid protein deposition, and inhibit excessive phosphorylation of the Tau protein. Research has shown that its structural analog, ABL, also suppresses the expression of COX-2 and NF-κB and alleviates Aβ23-35-induced learning and memory deficits in rats (Wang et al., 2008).

In the 5xFAD transgenic AD mouse model, OABL significantly reduced the immunofluorescence signal intensity of the NF-κB p-p65 subunit in both the cortex and hippocampus.This reduction occurred through modulation of the TLR4/NF-κB and p38 MAPK signaling pathways, and decreased the mRNA expression of pro-inflammatory cytokines such as TNF-α and IL-1β. It has also been demonstrated to promote the M1/M2 transformation of microglia, enhance the expression of arginase-1 (Arg-1) and IL-10, and suppress the production of TNF-α, PGE2, iNOS, and COX-2, thereby reducing the inflammatory response of the CNS and exerting potential neuroprotective effects (Tang et al., 2022).

#### 4.4.2 Berberine (BBR)

BBR is a naturally occurring isoquinoline alkaloid primarily derived from the roots, bark, and stems of various medicinal plants, such as the rhizomes of Coptis chinensis (Ye et al., 2009). It exhibits a wide range of pharmacological activities, including anti-inflammatory, cardioprotective, neuroprotective, antitumor, and antimalarial properties (Ma et al., 1999; Küpeli et al., 2002; Le Tran et al., 2003; Zheng et al., 2003; Kettmann et al., 2004; Račková et al., 2004; Letašiová et al., 2006). In the domain of AD research, BBR has demonstrated a variety of mechanisms of action, indicating its potential for therapeutic use.Recent studies have demonstrated that BBR can inhibit the production of pro-inflammatory cytokines, such as interleukin-6 (IL-6) and C-C motif chemokine ligand 2 (CCL2), in Aβ-stimulated primary microglia and BV-2 cell lines. Furthermore, it has been observed to downregulate the expression of COX-2 and iNOS. While the precise mechanisms through which BBR exerts its anti-inflammatory effects remain to be fully elucidated, current evidence suggests that it may do so primarily through the modulation of signaling pathways, including NF-κB, phosphoinositide 3-kinase (PI3K), and MAPK.

Wang and Zhang’s research in 2018 revealed that BBR demonstrated neuroprotective effects in preventing learning and memory deficits induced by traumatic brain injury. These effects were potentially attributable to the reduction of inflammation, oxidative stress, and neuronal apoptosis, as well as the modulation of the Sirt1/p38 MAPK signaling pathway (Wang and Zhang, 2018). In addition, BBR has demonstrated protective effects in rat models of heavy metal-induced neurotoxicity and AD-like pathology. Jia et al. (2012) utilized network pharmacology to identify cross-targets of BBR in AD and pinpointed the JNK–p38 MAPK signaling pathway as a critical regulatory pathway. Subsequent *in vitro* and *in vivo* experiments confirmed that BBR exerts its therapeutic effects in AD by activating autophagy, modulating the JNK–p38 MAPK signaling pathway to clear Aβ deposits, suppressing neuroinflammation, and promoting neuronal repair.

#### 4.4.3 Sesame oil (SO)

The Chinese herbal medicine Sesame is rich in sesame oil, which is the main natural source of sesamin. Sesamin has been demonstrated to possess a variety of pharmacological activities, including anti-inflammatory, antioxidant, antitumor, and cardiovascular protective effects (Jayaraj et al., 2020). SO has also been demonstrated to reduce monoamine oxidase (MAO) activity by inhibiting the production of nitric oxide (NO) and hydrogen peroxide (H_2_O_2_) in astrocytes. Given that MAO plays a critical role in the pathogenesis of NDDs, sesamol is considered to have significant potential in the prevention and treatment of CNS diseases.

Hou et al. (2003) demonstrated that SO significantly reduces NO production as well as iNOS mRNA and protein expression in LPS-stimulated BV-2 microglial cells. Furthermore, SO markedly inhibited the activation of p38 MAPK. The specific p38 MAPK inhibitor SB203580 also exhibited dose-dependent inhibition of NO production, further supporting the hypothesis that polyphenolic compounds capable of suppressing NO generation may exert neuroprotective effects.

In a related study, Wu et al. (2015) treated RAW 264.7 macrophages with sesamol followed by LPS stimulation to induce an inflammatory response. Their findings indicated that sesamol exhibited the capacity to impede NF-κB nuclear translocation and MAPK pathway activation, while concomitantly promoting the activation of AMP-activated protein kinase (AMPK). These findings suggest that sesamol improves inflammatory responses and oxidative stress damage by activating the AMPK and Nrf2 signaling pathways while inhibiting the NF-κB and MAPK pathways.

Mohamed et al. (2021) reported that SO significantly ameliorated AlCl_3_-induced learning and memory deficits in mice. It reduced AChE activity and Aβ levels, downregulated the expression of pro-inflammatory cytokines TNF-α and IL-1β, suppressed NF-κB and p38 MAPK signaling, and upregulated the expression of brain-derived neurotrophic factor (BDNF) and peroxisome proliferator-activated receptor gamma (PPAR-γ). These results suggest that SO alleviates neuroinflammation and oxidative stress damage by modulating the NF-κB/p38MAPK/BDNF/PPAR-γ signaling pathway, thereby contributing to the recovery of cognitive function and showing its potential value in the treatment of AD.

#### 4.4.4 Schisandrin A (SCH A)

SCH A is a bioactive lignan compound that has been isolated from Schisandra chinensis, a traditional Chinese medicinal herb. In recent years, SCH A has attracted growing scientific interest owing to its broad spectrum of pharmacological activities. It has been demonstrated to exert diverse biological effects, including anti-inflammatory, anticancer, hepatoprotective, antioxidant, neuroprotective, antidiabetic, and musculoskeletal protective properties (Choi, 2018; Cui et al., 2020; Kong et al., 2018; Meng et al., 2019; Jeong et al., 2019; Wang et al., 2014; Zhang et al., 2010). Notably, Schisandra and its active constituents have shown promising potential in the prevention and treatment of AD.

A series of experimental studies have demonstrated SCH A (10, 20, and 50 μM) suppresses the expression of NO, tumor necrosis factor-α (TNF-α), and IL-6 in LPS-stimulated BV-2 microglia and primary microglial cells, thus exerting anti-inflammatory properties. It mitigates microglia-mediated neuroinflammation by inhibiting key signaling pathways, such as TRAF6–IKKβ–NF-κB and JAK2–STAT3, thereby exerting neuroprotective effects (Song et al., 2016).

Furthermore, schisandrin has been shown to enhance neuronal viability in Aβ1–42-induced SH-SY5Y cell models of AD through activation of the PI3K/Akt signaling pathway, thus exerting protective effects (Zhao et al., 2019). Jia et al. (2024) reported that SCH A significantly reduces oxidative stress response and downregulates inflammatory cytokine expression in cells induced by Aβ25-35, while also increasing the p-ERK1/2 to ERK1/2 ratio, indicating that its underlying mechanism may involve activation of the ERK/MAPK pathway.

Further research by Kwon et al. (2018) using an *in vitro* RAW 264.7 macrophage model demonstrated that SCH A attenuates LPS-induced inflammation and oxidative stress by activating the Nrf2/HO-1 signaling pathway, while concurrently suppressing the NF-κB, MAPK, and PI3K/Akt pathways. Among these, SCH A pretreatment markedly inhibited the phosphorylation of ERK, JNK, and p38 MAPK, providing further evidence of its multi-target anti-inflammatory and antioxidant effects.

#### 4.4.5 Crocin (CRO)

Crocin (CRO) is a natural carotenoid that is found in high concentrations in the stigmas of saffron (Crocus sativus) and the fruits of gardenia (Gardenia jasminoides) (Liu T. et al., 2020). Extensive *in vitro*, *in vivo*, and clinical studies have demonstrated that CRO exerts beneficial effects across multiple organ systems, including the nervous, immune, cardiovascular, gastrointestinal, reproductive, and endocrine systems (Khorasany and Hosseinzadeh, 2016).

Research indicates that CRO exerts significant memory-enhancing effects, which are partly attributed to its anti-inflammatory properties and modulation of the ERK/MAPK signaling pathway. In a D-galactose-induced aging model, CRO improves cognitive function via its anti-glycation and antioxidant activities, thereby suppressing the expression of neuroinflammatory mediators (e.g., IL-1β, TNF-α, and NF-κB) and activating the PI3K/Akt and ERK/MAPK signaling pathways (Adabizadeh et al., 2019; Heidari et al., 2017; Looti Bashiyan et al., 2021).

Furthermore, the use of CRO has been shown to markedly decrease total tau protein levels and phosphorylation, suppresses β- and γ-secretase activities, and reduces the deposition of Aβ precursor protein (AβPP) accumulation in AD models by inhibiting ERK1/2 kinase activity (Chalatsa et al., 2019). Another study shows that CRO mitigates acrolein-induced neurotoxicity, potentially through the attenuation of oxidative stress via the ERK/MAPK pathway, thus delaying the progression of NDDs (Rashedinia et al., 2015).

#### 4.4.6 Ginsenosides Re, Rg1, and Rg2

Ginsenosides Re, Rg1, and Rg2 are the major triol-type natural saponins in ginseng and represent the principal active constituents of this traditional Chinese medicinal herb. These compounds have been demonstrated to exert a variety of pharmacological effects, including the enhancement of cognitive function, the inhibition of apoptosis, and the exertion of neuroprotective activities (Li et al., 2020). Among them, ginsenoside Re is a pivotal component (Shi et al., 2019) and remains the most extensively investigated ginsenoside to date. It has been demonstrated to possess antioxidant and anti-inflammatory properties, suppressing the production of IL-6, tumor necrosis TNF-α, and NO in microglial cells without impairing cellular viability (Lee et al., 2020; Lee I-A. et al., 2012). The reduction in the release of pro-inflammatory and neurotoxic mediators from microglia has been demonstrated to provide a protective effect on hippocampal neurons (Madhi et al., 2021). Furthermore, ginsenoside Re has been shown to attenuate neuroinflammation progression by inhibiting LPS-induced MAPK phosphorylation (Lee K-W. et al., 2012).

Among the diverse ginsenosides, Rg1 demonstrates notable neuroprotective benefits, especially in NDDs such as AD and PD. Hu et al. (2011) demonstrated that ginsenoside Rg1 suppresses LPS-induced microglial activation via downregulation of Iba-1 and iNOS expression. Furthermore, Rg1 effectively inhibits the phosphorylation of p38 MAPK, ERK1/2, and JNK, and prevents the degradation of IκB as well as the nuclear translocation of the NF-κB p65 subunit. Rg1 attenuates LPS-induced inflammatory responses by activating the phospholipase C-γ1 signaling pathway in mouse BV-2 microglia. It inhibits the phosphorylation of p38 MAPK, IκB-α, CREB, and ERK1/2, significantly reduces NF-κB expression, and decreases the production of pro-inflammatory cytokines, including TNF-α, IL-1β, iNOS, and COX-2 (Zong et al., 2012). The neuroprotective effect of ginsenoside Rg1 against LPS-induced neuronal degeneration in rats is mediated via the glucocorticoid receptor, involving inhibition of the p38 MAPK signaling pathway to suppress LPS-induced inflammation in midbrain dopaminergic neuronal microglia (Sun et al., 2016).

Treatment with ginsenoside Rg2 significantly elevates the ratios of phosphorylated ERK to total ERK (p-ERK/ERK) and phosphorylated MAPK to total MAPK (p-MAPK/MAPK) in the brain tissue of 3xTg-AD mice, thereby mitigating neurovascular damage in this AD model (Ye et al., 2023).

#### 4.4.7 Black ginseng extract (BGE)

Panax ginseng, which is more commonly referred to as Korean ginseng, contains primary active components including ginsenosides (-Rg3, -Rg5, and -Rk1), polysaccharides, and phenolic compounds, with particularly high concentrations in Korean BGE (Metwaly et al., 2019). Research has demonstrated that extracts of Korean BGE, when administered as an ethanol solution, have been shown to attenuate neuroinflammation by inhibiting the NF-κB and MAPK signaling pathways in LPS-stimulated BV2 microglia. This attenuation is achieved via a Toll-like receptor 4 (TLR4)-MyD88-dependent mechanism (Kim et al., 2023). Furthermore, BGE has been demonstrated to significantly enhance cognitive function in the 5xFAD AD mouse model, concomitant with reduced Aβ accumulation in the frontal cortex and hippocampus (Ha et al., 2025). BGE has been demonstrated to suppress the activation of microglia and astrocytes, as well as to downregulate pro-inflammatory cytokines, including IL-6 and tumor necrosis factor-alpha (TNF-α), along with the expression of enzymes such as COX-2 and iNOS. Further studies have demonstrated that BGE reduces Aβ plaque deposition via activation of the nuclear factor erythroid 2-related factor 2 (Nrf2)/heme oxygenase-1 (HO-1) pathway and suppresses p38 MAPK, NF-κB, and STAT3 signaling pathways, as well as NLRP3 inflammasome activation, thus protecting cognitive function in 5xFAD mice and highlighting its therapeutic potential in AD.

#### 4.4.8 Esculetin (ESC)

Esculetin is a natural dihydroxy coumarin; it is mainly extracted from twig skin and the trunk bark of the Chinese herbal medicine Fraxinus rhynchophylla Hance.Natural coumarin derivatives have demonstrated anti-inflammatory effects through various inflammatory signaling pathways, including TLRs, JAK/STAT, inflammasomes, MAPK, NF-κB, and TGF-β/SMAD. Possesses antioxidant (Wang et al., 2011), antiinflammatory (Kirsch et al., 2016), antiapoptotic (Kim et al., 2015), anticancer (Pinto and Silva, 2017), antidiabetic (Li et al., 2017), neuroprotective (Delogu and Matos, 2017), and cardiovascular protective activities (Najmanová et al., 2015). Pruccoli et al. (2020) demonstrated the ability of ESC to prevent and counteract ROS formation in neuronal SH-SY5Y cells, suggesting its profile as a bifunctional antioxidant. In particular, ESC increased the resistance of the SH-SY5Y cells against OS through the activation of Nrf2 and increase of GSH. In similar experimental conditions, ESC could also protect the SH-SY5Y cells from the OS and neuronal death evoked by oligomers of Aβ1-42 peptides. Further, the use of the inhibitors PD98059 and LY294002 also showed that Erk1/2 and Akt signaling pathways were involved in the neuroprotection mediated by ESC.

ESC, a common coumarin derivative, was reported by Song et al. (2018) to exhibit protective potential against diabetic nephropathy (DN). In this study, a diabetic mouse model was established in 6-week-old male C57BL/6J mice by a single intravenous injection of streptozotocin (STZ, 30 mg/kg). Two weeks after STZ injection, the mice received intravenous administration of ESC at doses of 5, 10, or 20 mg/kg for an additional 2 weeks. The results demonstrated that ESC markedly suppressed STZ-induced renal expression of AP-1, p-p38 MAPK, and p-JNK, while upregulating p-ERK1/2. These findings suggest that ESC may alleviate experimental DN-associated cognitive impairment through modulation of the MAPK signaling pathway, exerting both antioxidative and anti-inflammatory effects.

## 5 Discussion

Alzheimer’s disease (AD) is a complex chronic neurodegenerative disorder characterized by multiple pathological processes, including β-amyloid (Aβ) deposition, tau hyperphosphorylation, neuroinflammation, and oxidative stress. Given the limited efficacy and adverse effects of current therapies, natural products, owing to their multi-target actions and relative safety, have attracted increasing attention as promising candidates for AD prevention and treatment.

This review systematically summarizes recent progress on various classes of natural compounds in AD research, including flavonoids, phenolics, saponins, terpenoids, and alkaloids. Representative compounds such as 1,6-O,O-diacetylbritannilactone, berberine, sesamol, schisandrin A, crocin, ginsenosides, and coumarins have demonstrated potential neuroprotective effects by improving cognitive performance, alleviating neuroinflammation, reducing oxidative stress, and inhibiting neuronal apoptosis. Accumulating evidence suggests that these compounds exert their beneficial effects mainly through the modulation of signaling pathways such as NF-κB, MAPK, PI3K/Akt, and Nrf2/HO-1, thereby interfering with key pathological events of AD. Moreover, both *in vitro* and *in vivo* studies have shown that natural products significantly suppress neuroinflammatory responses and ameliorate cognitive impairments in AD animal models.

Despite these advances, current research models remain limited. Most animal studies rely on short-term acute dosing and lack long-term administration protocols, whereas the chronic and progressive nature of AD suggests that therapeutic efficacy may depend on sustained exposure. In addition, systematic toxicological evaluations of candidate compounds are insufficient, particularly concerning blood-brain barrier (BBB) permeability, organ-specific toxicity, and long-term safety. Furthermore, the intrinsic issues of low bioavailability and complex *in vivo* metabolism substantially restrict their clinical translation. The potential of combining natural products with existing drugs also remains largely unexplored.

To facilitate the effective translation of natural compounds from bench to bedside, it is essential to establish experimental systems that align with translational medicine standards. These include long-term pharmacodynamic evaluations in chronic disease models and comprehensive preclinical safety assessments in accordance with ICH guidelines. More importantly, high-quality clinical studies that comply with international standards—such as multicenter, randomized, double-blind trials and biomarker-based validation studies—are urgently required to confirm the clinical efficacy of natural bioactive compounds and meet regulatory approval requirements. Currently, several promising natural compounds (e.g., huperzine A derivatives, ginkgolide-related components) are at different stages of development, and systematic investigations are expected to accelerate the clinical translation of more AD candidate drugs with therapeutic potential.

In conclusion, natural products, by virtue of their multi-target mechanisms and relatively low toxicity, represent a promising avenue for AD therapy. Future studies should focus on systematic evaluations of long-term efficacy and safety, optimization of drug delivery strategies, and implementation of high-quality clinical trials, thereby laying a solid foundation for their eventual clinical translation.
