# Correction: Research progress on plant-derived natural compounds regulating the MAPK signaling pathway for the prevention and therapy of Alzheimer’s disease

**DOI:** 10.3389/fphar.2025.1710820

**Published:** 2025-09-30

**Authors:** Xinyue Zhang, Shujia Huang, Hongfeng Xu, Yanan Hu, Ling Gao

**Affiliations:** Changchun University of Chinese Medicine, Changchun, China

**Keywords:** Alzheimer’s disease, neurodegenerative disease, natural compound, mitogen-activatedprotein kinase, research progress

All authors were erroneously assigned to the **affiliation** “The Third Affiliated Hospital of Changchun University of Chinese Medicine, Changchun, China”. This affiliation has now been removed and the correct affiliation has now been added as: Changchun University of Chinese Medicine, Changchun, China.

The original article has been updated.

